# The surgical experience of current non-surgeons gained at medical school: a survey analysis with implications for teaching today’s students

**DOI:** 10.1186/s12909-015-0466-3

**Published:** 2015-10-27

**Authors:** Sabine Zundel, Adrian Meder, Stephan Zipfel, Anne Herrmann-Werner

**Affiliations:** 1 Department of Pediatric Surgery, University Hospital of Tuebingen, Hoppe-Seyler-Str. 3, 72076 Tuebingen, Germany; 2 Berufsgenossenschaftliche Unfallklinik Tuebingen, Tuebingen, Germany; 3 Department of Psychosomatic Medicine and Medical Faculty, University Hospital of Tuebingen, Tuebingen, Germany; 4 Department of Psychosomatic Medicine and Medical Faculty Tuebingen, Multidisciplinary Skills Lab “DocLab”, University Hospital of Tuebingen, Tuebingen, Germany

**Keywords:** Clerkship, Learning objectives, Skills, Surgery Education

## Abstract

**Background:**

It is unknown what aspects of undergraduate surgical curricula are useful for future non-surgeons. We aimed to define relevant, enduring learning achievements for this subgroup to enable student-centered teaching.

**Methods:**

An online questionnaire using open ended questions was distributed to physicians of non-surgical specialties at the University Hospital of Tuebingen, Germany and its associated teaching hospitals. Participants were asked to describe knowledge and skills that endured from their surgical clerkship and which of these are used in daily practice. Textual responses were initially coded using content analysis and the frequency of recurrent categories was calculated.

**Results:**

Sixty-seven of 153 questionnaires were returned; participants belonged to six different non-surgical specialties and had received their training at 22 different medical schools. Sustaining learning achievements included basic skills (suturing and working under sterile conditions), learning about professionalism and appreciating working conditions in surgery. Two learning techniques were valued: witnessing of rare cases or complications and working autonomously.

**Conclusion:**

Integration of our findings in undergraduate surgical teaching may focus teaching on students’ interests and improve surgical teaching.

## Background

Many departments report significant difficulties delivering effective surgical teaching and students describe dated curricula [1]. Results from student surveys found that the lack of clearly defined and articulated learning objectives is one reason for the perceived deficiency [2]. There are, however, few studies evaluating learning objectives for undergraduate surgical education and calls for their reassessment have been published [3]. Past studies have focused on students’ perception of the learning experience [4] or the clerkships’ learning objectives to foster interest in surgery [5–7]. Data on relevant learning objectives for students who later work in a non-surgical specialty has not been published previously. Since the majority of students undergoing undergraduate surgical training will not go on to work in a surgical specialty in the future it is especially their interests that need to be considered. If valued learning achievements are known, they can be heeded, made explicit, and satisfaction with surgical training may be improved.

To develop relevant learning objectives for an undergraduate surgical curriculum, we analyzed learning achievements valued from a group hitherto neglected: With an online-questionnaire, we asked current non-surgeons: “What knowledge, learned during their surgical clerkship endures, and which skills are applied regularly?”

## Methods

An online, web based instrument was used. The questionnaire was pilot tested on the members of a current Master of Medical Education class in Bern, Switzerland (physicians, working in non-surgical specialties *n* = 12). Pretest data suggested that there is little decline in recollection of remarkable surgical experience and participants were able to connect their knowledge with medical school experience regardless of time since medical school training. The target population was defined as physicians of non-surgical specialties, independent of their age or professional experience. Ethical approval was obtained by the ethical committee of the department of Medicine at the University of Tuebingen (Project No.: 432/2013A).

The study took place at the University Hospital of Tuebingen and associated teaching hospitals. The offices of the non-surgical departments, namely: Anesthesiology, Internal Medicine, Neurology, Pediatrics, Psychiatry, and Radiology were contacted by the authors. The study was described and measures for privacy protection were explained. All departments agreed to participate and supplied email contact data of their physicians. The single exclusion criterion for participation was post-graduate experience in a surgical specialty.

Email addresses of 153 physicians working in non-surgical discipline were obtained. Emails were sent with an invitation to participate in the study, a link to the questionnaire was included. A second email with a reminder was sent one week after the initial email if the questionnaire had not been opened. IP addresses were not recorded, the software does not allow tracing of completed questionnaires to the originating email addresses. Personal data of the participants was neither requested nor saved if acquired by chance (direct inquiries by participants). All participants agreed to citation of statements.

Data collection took place in June 2013. The online questionnaire was conducted and distributed using the web based tool “oFb - der onlineFragebogen” from SoSci Survey®. The questionnaire started with questions concerning gender, age, specialty, medical school, time interval since surgical education and postgraduate surgical experience. Participants who checked “yes” for “postgraduate surgical experience” were excluded from the analysis. These demographic questions were followed by three open questions concerning experiences remembered as relevant, valued learning achievements and a specification of which of these the participants still use in their daily work. An English translation of the original questionnaire can be found in Table 1.

**Table 1:** Questionnaire (translated from German)

Topic	Question
Gender	Selection: Female - Male
Age	Please state your current age in years.
Specialty	What specialty are you currently working in?
Postgraduate surgical experience	Did you ever work in a surgical department? Maybe in form of a rotation?
Interval	How long does your surgical clerkship date back? Please state the space of time in years.
Location	Please name the medical school where you undertook your surgical clerkship
Remarkable, memorable experience	Which specific experience do you remember and can recall as outstanding or relevant?
Valued learning achievements	What are your most valued learning achievements from your time as a surgical clerk?
Applied skills	Please name knowledge and skills you still use during your current work.

## Data analysis

Descriptive data was recorded to characterize the sample population. Participant texts were read and coded by SM and AM, in accordance with Mayring’s recommendations [8]. Each author independently identified learning objectives. After this first analysis, the findings were discussed by all authors and categories were phrased. Having established the categories, the authors returned to the original data and rechecked the texts. Care was taken, that all categories accurately reflect the participants’ statements. Categories were again discussed within the team and adjusted if necessary. When discovering a new learning achievement during this second analysis of the texts, the process mentioned above was repeated. As soon as no new achievements were discovered and all authors had verified the extracted categories as participants’ beliefs’; the second phase of the data analysis was started: The frequency of each category was calculated. Exemplary citations were extracted from the texts for every achievement. The citations were used to recheck the authenticity of the categories. Learning achievements seldom mentioned were included into the analysis if the texts suggested active reflection about the individual achievement or if participants gave a convincing reason for the importance of the achievement. Disagreement between the authors about categories or decision about inclusion was resolved by consensus.

## Results

Sixty-seven questionnaires were returned, this represents a response rate of 43 %. There were 41 female (61 %) and 26 male participants. One set of answers was excluded from the data analysis because the participant had worked in surgery for 7 years. The median age of participants was 35 years with a standard deviation of 5.6 years. As expected, the age of participants related proportionally to the time interval since clerkship experience. The time-lag since the surgical experiences was a median of 8 years with a standard deviation of 5.4 years.

Internal Medicine and Pediatrics were the most common specialties. The detailed list of the specialties is shown in Table 2. The majority of participants (*n* = 30) received their surgical training at the University of Tuebingen, the place where the study was undertaken. The remaining group is heterogeneous. Fifteen of the 36 medical schools within Germany were represented with one to three participants. International experience was gathered at Pretoria, South Africa and Indianapolis, USA and at three locations in Switzerland. A detailed list of locations can be obtained from the author.

**Table 2:** Specialties of participants

Specialties	Number of participants
Anesthesiology	8
Internal Medicine	22
Neurology	6
Pediatrics	17
Psychiatry	7 (one exclusion due to postgraduate surgical experience)
Radiology	7

The analysis resulted in five categories of learning achievements. These, the frequency of answers they were found in, and exemplary quotes, taken from a range of respondents are displayed in Table 3. Namely, valued learning achievements were:

**Table 3:** Learning objectives and exemplary citations

	Learning objective	Exemplary Citations
1	Practical skills: Suturing and working under sterile conditions *n* = 25	• I can close a laceration • At least I now have rudimentary skills on how to suture • I know how to suture and tie a surgical knot which helps after putting in a Sheldon catheter. • How to perform surgical hand scrubs • How to move when scrubbed • The rules of scrubbing
2	Professional Behavior: *n* = 16	
	• Relevance of team functioning and team communication	• The importance of good communication for a team • Communication is crucial for the team • I saw a good example of cooperation between different specialties. • Sometimes it’s better not to speak one’s mind • the OR is no place for democracy, the one who is in charge must decide
	• Management of critical incidents	• Being disciplined triggers professionalism • I learned the relevance of keeping calm in critical situations • Keeping calm is most important
	• Altruism/Self-care	• Keep going and put personal matters aside •Making sure one eats and drinks enough • Coping with fainting
	• Work ethics	• Importance of accuracy for patient safety • Concentration is all important • If a surgeon works sloppily, complications arise and the patient pays
3	Working conditions *n* = 15	• There is an enormous amount of pressure on surgeons • Standing for long hours without breaks • It’s such hard work • The work is physically exhausting
4	Highlights *n* = 12	• I remember individual procedures where a complication occurred • I witnessed a very bloody procedure on a ruptured aneurysm. • A traumatic penis rupture looked awful.
5	Working autonomously under supervision *n* = 7	• I was allowed to do an orchidopexy, I will never forget the indication for this procedure. • I did an implant removal and experienced how different tissues feel.

[n] = the number of participants who recognized this as a learning objective

1: basic practical skills (the ability to suture and working under sterile conditions), 2: professional behavior, 3: working conditions within the operating room (OR), 4: recollection of specific complications or rare clinical cases and 5: students’ active participation under direct supervision.

All learning objectives were found equally in male and female answers. Special interest was taken regarding the learning objectives “working conditions” and “professional behavior” since a gender difference was expected to be more likely in these areas [9]. No difference could be reported, however, both genders contributed answers to these learning objectives.

### Category 1: basic skills

Suturing was the skill that most frequently appeared in the answers given. Seventeen participants (26 %) rated this as the most important item they learned during their surgical training. Comments displayed pride in mastering the skill and the ability to suture seemed to be a positive characteristic within non-surgical specialties. Working under sterile conditions and scrubbing correctly was significant for 8 participants (12 %).

### Category 2: professional behavior

Within 16 statements (24 %), a learning achievement concerning professional behavior was identified. Specifically these were:

#### Team function

Participants vividly recalled events that taught them the relevance of team functioning and team communication. Answers included examples of good inter-professional cooperation and concrete examples for ways to behave.

#### Management of critical incidents

Repeatedly the management of critical incidents, particularly staying calm in stressful situations was named as an important learning achievement. Comments suggest that role-modeling played a major role in teaching this skill.

#### Altruism/Self-care

Two comments explicitly mentioned personal sacrifice and self-care. A significant number of answers additionally commented on little possibility for self-care in the field and an exceedingly high necessity for altruism.

#### Work ethics

Three participants reported on enlightenment related to the importance of accuracy and its effect on patient safety.

### Category 3: working conditions

Fifteen participants named the realization regarding the strenuousness of working in the OR as an important memory. 10 participants named the bad working conditions as significant recollections. Comments included: windowless rooms, long periods of standing upright, long duty hours and short-term changes of the OR schedule. Most of these comments were accompanied by declarations that the experiences lead to consciously deciding not to specialize in surgery.

### Category 4: highlights

Twelve answers (18 %) syconsisted of detailed memories of specific events the participants witnessed. These recollections represented unique clinical cases; they included a liver transplantation into a two year old child, a traumatic amputation of an arm, the birth of twins by caesarean section and a traumatic penis rupture.

### Category 5: students working autonomously under direct supervision

Seven participants named procedures and incidents, where they participated autonomously and were able to take responsibility for certain procedures. Answers indicated that these students were highly involved in their work and the learning achievement includes factual knowledge, skills and professionalism.

## Discussion

The questionnaires yielded multifaceted data on the subject of learning achievements for future non-surgeons. No similar summary has been published previously. The collected data may aid surgical teachers in choosing learning objectives and guide them towards increased student-centeredness.

### Discussion of methodology

Online questionnaires, as a methodology to obtain qualitative data, proved easily feasible. The procedure had a number of strength: distribution was easy to handle and enabled physicians to participate with minimal effort. According to the literature, e-mail survey response rates range from 19 to 61 %. Potential influences on response rates are: survey length, design issues, research affiliation and compensation [10], which were taken into account when designing our study. The questionnaire was kept short and the invitation email contained information on the anticipated duration of participation. The sample size was limited to the University Hospital of Tuebingen to allow affiliation. These efforts accounted to a satisfactory response rate of 43 %.

The methodology proved adequate to obtain a broad view of learning achievements. The opportunity to provide open-text responses anonymously encouraged free expression in the answers given. The diversity of answers, length of comments and detailed descriptions exceeded the authors’ expectations. Questionnaire surveys however do not allow for exploration of responses. Individual interviews or focus groups would have been superior. These options were discarded in favor of increasing the sample size and for feasibility reasons. A further possible confounder of our method of analysis is the wide variety of possible interpretations and the possibility of missing nuanced data. An attempt to counteract this was made by examining the data repeatedly and using an iterative procedure of analysis.

### Discussion of sample population and generalizability

Data was collected from participants representing 6 different non-surgical specialties; the undergraduate surgical education was undertaken at 22 different Medical schools. Current surgeons were not included since it was suspected that they would not be able to differentiate whether an experience was gained at the end of medical school or at the start of professional training.

Comments representing each category were found within all specialties and all medical schools. Because of this accordance we conclude that the data is representative for non-surgical physicians in Germany. The great variety of included medical schools further allows generalizability. If the data had been restricted to a small number of medical schools, clerkship experience might have represented individual local conditions. However, with 67 respondents, the number of participants is limited and data might have been missed due to the relatively small sample size.

Sixty-one percent of the participants of our survey were female, a review of German figures from the federal statistical office (2012) demonstrate that 62 % of medical first-years are female. The sample population is therefore representative for current Medical school students and the future workforce.

Anesthetists, working in the operating room (OR) regularly, might have an altered perspective. Therefore, anesthetists’ answers were tested for differences and the analysis was repeated on their data individually. No differences were identified.

Recall bias needs to be discussed as a confounder. The literature suggests, that the recall accuracy of surveys is related to a number of characteristics: appropriate setting, time provided to answer questions, introduction and motivation to participate [11]. Choosing an online questionnaire and allowing affiliation we limited this confounder as much as possible. The time interval between experience and recollection did not present itself as a confounder in the pretest, and this was confirmed in the data. Answers of older participants were similarly detailed, vivid and diverse if compared to the answers of younger participants.

### Discussion of results

Five categories for major learning achievements were generated from the data. Basic skills (suturing and working under sterile conditions) was the most valued learned item. Both skills are being taught in simulation settings at most German medical schools today. Many studies emphasize the importance of simulation. A recently published study demonstrated significant improvement in surgical skills by the running of a workshop [12]. Nevertheless, studies on workplace based education show that next to practical competence; students need to establish a sense of professional identity that includes confidence and motivation [13].

Sixteen out of 66 participants (24 %) in our study rated professional behavior as their most important learning issue. Fernando at al. published data on students’ expected learning objectives during surgical experience. Fact acquisition; revision of basic science (anatomy, physiology and pathology) and learning about surgical conditions were found to be the most often named items. “Only 11 % of the respondents considered staff roles and interactions […] important learning objectives” [14]. This comparison suggests that students’ pre-experience expectations and physicians post-experience acknowledgements differ greatly.

The insight summarized within the category ‘professionalism’ was further divided into subheadings. This might guide a surgical teacher in his choice of learning objectives concerning professionalism. Using the environment of the OR to teach about teamwork and the management of critical incidents is somewhat obvious. Statements regarding ‘self-care/altruism’ and ‘work ethics’ were mentioned by a small number of participants. They were nevertheless included as subcategories because they present important issues which often do not have a strict allocation into any field of medical teaching. Our data shows that the OR is one place where students can learn about these aspects.

When including professional behavior as a learning objective, surgical teachers need to reflect whether they teach what they are aiming to teach. In 2013 Bennett et al. found that “the meaning students make of the real-world performance of professional roles […] remain unclear’ [15]. In 2011 Lamiani et al. reported on the hidden curriculum and how some of the messages conveyed may conflict with formally taught learning objectives [16]. Principles of teaching professionalism in medicine have been published by Cruess et al. in 2006. Bennett and Lamiani’s findings suggest that this knowledge needs to be further disseminated among medical teachers, becoming part of faculty development programs.

Our participants’ observations on the working conditions of surgeons, especially the strenuousness of the job is in line with findings from other studies and was shown to influence career choices. Prolonged working hours during the surgical training period was found to be the only significant predictor for not choosing general surgery in a study published in 2011 [17].

The statements summarized into category 4, the detailed recollection of very specific events and the ability to retrieve this factual knowledge, for example the management of precise cases, can be explained by the emotional involvement of the student in these cases. The impact of emotions on learning is subject to current studies and has been the base of recently developed conceptual frameworks for adult learning [18]. The OR regularly presents students with emotionally demanding situations. This should be reflected by surgical educators and its implications should be recognized and used. Outstanding experiences need to be recognized as relevant teaching opportunities. Teaching factual knowledge might be enhanced by utilizing the emotional involvement of students. These learning achievements contained factual knowledge, professional behavior and communication skills.

This positive learning opportunity might be further enriched by taking in consideration the implications that can be extracted from learning objective no. 5 (working autonomously under supervision). Participants vividly recalled incidents where they were allowed to perform tasks themselves. Students are well aware of the importance of active participation as a tool for better learning [19]. The implications of category 4 and 5 cannot be limited to students not wanting to become surgeons. These finding identify opportunities to improve teaching in the OR in general. Studies have shown that active participation increases the interest in surgery [6].

## Conclusion

We were able to define learning achievements - dating back to their undergraduate studies - valued by non-surgeons in their later career. Practicing basic skills and learning about professional behavior was highly valued. Exceptional cases and active participation were found to encourage learning. The inclusion of the generated categories and methods into surgical teaching might focus the teaching on students’ interests and improve evaluations. Further research needs to elaborate these findings further.

## Abbreviation

OR: Operating room
